# A Deformed Exponential Statistical Manifold

**DOI:** 10.3390/e21050496

**Published:** 2019-05-15

**Authors:** Francisca Leidmar Josué Vieira, Luiza Helena Félix de Andrade, Rui Facundo Vigelis, Charles Casimiro Cavalcante

**Affiliations:** 1 Departamento de Matemática, Universidade Regional do Cariri, Juazeiro do Norte-CE 63041-145, Brazil; 2 Departamento de Ciências Naturais, Matemática e Estatística, Universidade Federal Rural do Semi-Árido, Mossoró-RN 59625-900, Brazil; 3 Curso de Engenharia de Computação, Campus Sobral, Universidade Federal do Ceará, Sobral-CE 62042-280, Brazil; 4 Departamento de Engenharia de Teleinformática, Universidade Federal do Ceará, Fortaleza-CE 60020-181, Brazil

**Keywords:** deformed exponential manifold, statistical manifold, *φ*-family, information geometry, exponential arcs

## Abstract

Consider μ a probability measure and $P_{\mu }$ the set of μ-equivalent strictly positive probability densities. To endow $P_{\mu }$ with a structure of a $C^{\infty }$-Banach manifold we use the φ-connection by an open arc, where φ is a deformed exponential function which assumes zero until a certain point and from then on is strictly increasing. This deformed exponential function has as particular cases the *q*-deformed exponential and κ-exponential functions. Moreover, we find the tangent space of $P_{\mu }$ at a point *p*, and as a consequence the tangent bundle of $P_{\mu }$. We define a divergence using the *q*-exponential function and we prove that this divergence is related to the *q*-divergence already known from the literature. We also show that *q*-exponential and κ-exponential functions can be used to generalize of Rényi divergence.

## 1. Introduction

Let $P_{\mu }$ be the set of μ-equivalent strictly positive probability densities, where μ is a given probability measure. In order to build a structure to $P_{\mu }$, Amari considered the parametric case, where the construction depends on a parameter belonging to the Euclidean space [1,2]. The case of non-parametric statistical models was initially studied by Pistone and Sempi [3]. In this case, $P_{\mu }$ was equipped with a structure of a $C^{\infty }$-Banach manifold using the Orlicz space associated to an Orlicz function. In a later work [4], Pistone and Cena proved that the probability distribution *z* belongs to the maximal exponential model to the probability distribution *p*, if and only if, *z* is connected to *p* by an open exponential arc. Moreover, the new manifold structure obtained from the connection by an open exponential arc is equivalent to the one defined in [3,5]. Results involving conditions connecting two probability densities by an open exponential arc were recently studied in [6].

The deformed exponential function was first introduced by Naudts in [7] and studied in more details later in [8,9]. In [10], the authors propose a generalization for the exponential family $E_{p}$, based in the replacement of the exponential function exp by a deformed exponential function φ. It is then proposed a φ-family of probability distributions denoted by $F_{c}^{\varphi }$, with p=φ(c). The described family was modeled on Musielak–Orlicz spaces and a Banach manifold structure to $P_{\mu }$ is obtained. As a consequence of such model, a more general form of the Kullback–Leibler divergence was obtained and called φ-divergence. Furthermore, the arcs for the deformed exponential function were investigated and it was provided the necessary and sufficient conditions to connect by a φ-arc any two probability distributions [11]. This result was generalized later by [12,13]. A generalization to exponential arcs was defined in [14] and it also proved that the probability distribution *z* belongs to the φ-family $F_{c}^{\varphi }$ if, and only if, *z* is connected to *p* by an open φ-arc.

An example of deformed exponential function is the *q*-exponential one that it was used by Loaiza and Quiceno [15] to define an atlas modeled on essentially bounded function spaces. The charts for the given atlas are defined in terms of connections by an one-dimensional *q*-exponential model and of the *q*-deformations of cumulant maps [4]. Moreover, using equivalence class it was constructed the tangent space and the tangent bundle.

In this paper we endow $P_{\mu }$ with a structure of a $C^{\infty }$-Banach manifold using a deformed exponential function. This deformed exponential function has zero value until a certain point and from then on has the behaviour similar to the “classical” exponential function, which is strictly increasing. Particular cases of that function are: *q*-deformed exponential and κ-exponential. In order to build this structure, as in [15], we divide $P_{\mu }$ into equivalence classes using the connection provided by generalized exponential arcs as defined in [14]. Also, we define a set $A_{c}^{\varphi }$, that is the connected component of $P_{\mu }$ and will be the generalized φ-family of probability distributions. Moreover, by means of the derivative of the transition map, we find the tangent space and, consequently, the tangent bundle. In addition, we define a divergence using the *q*-exponential function which is related with the *q*-divergence defined in [15]. Finally, we show that the κ-exponential and *q*-exponential functions can be used in the generalization of Rényi’s divergence.

The rest of the paper is organized as follows. In Section 2 we revisit some important results about the *q*-exponential statistical manifold and provide a brief introduction about Musielak–Orlicz spaces. In Section 3, we have our main results. We discuss generalized open exponential arcs and build generalized φ-families of probability distributions. Alterwards, in Section 4, we find the derivative of the transition map and, as a consequence, the tangent space and tangent bundle. Moreover, in Section 5 we define a divergence using the *q*-exponential function and we use those results to prove that the *q*-exponential and κ-exponential functions can be used to generalize Rényi’s divergence. Finally, in Section 6 our conclusions and future perspectives are stated.

## 2. Background and Preliminary Results

The deformed exponential function that we will use to equip $P_{\mu }$ with a structure of a $C^{\infty }$-Banach manifold has as a particular case the *q*-exponential function and the parametrization domain is obtained from a Musielak–Orlicz space. For this reason, the purpose of this section is to make a brief presentation of the results involving the *q*-exponential manifold and the Musielak–Orlicz spaces.

### 2.1. A q-Exponential Statistical Banach Manifold

In the same way as in [15], we consider (T,Σ,μ) a probability space and q∈(0,1). The *q*-deformed exponential function is given by [16]

(1) $$e_{q}^{x}={\left(1+\left(1-q\right)x\right)}^{1/(1-q)},\;\mathrm{where}\;\;\frac{-1}{1-q}\leq x.$$

**Definition** **1.** *We say that $p,\;z\in P_{\mu }$ are connected by an one-dimensional q-exponential model if there exists $r\in P_{\mu }$, $u\in L^{\infty }\left(r.\mu \right)$, a real function of a real variable ψ and δ>0, such that for all t∈(−δ,δ) the function f defined by*

(2) $$f\left(t\right)=e_{q}^{tu{⊖}_{q}\psi \left(t\right)}r,$$

*satisfies that there are $t_{0},\;t_{1}\in \left(-\delta ,\delta \right)$, with $f(t_{0})=p$, $f(t_{1})=z$ and $tu{⊝}_{q}\psi \left(t\right):=\frac{tu-\psi (t)}{tu+(1-q)\psi (t)}$, for $\psi \left(t\right)\neq {\left(q-1\right)}^{-1}$.*

Consider the following partition of $P_{\mu }$ into equivalence classes: $p,\;z\in P_{\mu }$ are related ($p{∼}_{q}z$) if and only if there exists an one-dimensional *q*-exponential model connecting *p* and *z*, according to Equation (2). As a consequence, the measures p.μ and z.μ are equivalent and the essentially bounded function spaces $L^{\infty }\left(p.\mu \right)$ and $L^{\infty }\left(z.\mu \right)$ are equal.

We need to define a family of *q*-deformations of the moment-generating functional denoted by $M_{p}^{q}$, it means,

$$M_{p}^{q}:D_{M_{p}^{q}}\to \left[0,\infty \right],$$

$$M_{p}^{q}\left(u\right)=\int_{T}e_{q}^{\left(u\right)}d\mu ,$$

where

$$D_{M_{p}^{q}}=\left\{u\in L^{\infty }\left(p.\mu \right);\frac{-1}{1-q}<u,\;\int_{T}e_{q}^{\left(u\right)}d\mu <\infty \right\}.$$

Also, we define a family of cumulant generating functional

$$K_{p}^{q}:B_{p,\infty }\left(0,1\right)\to \left[0,\infty \right]$$

where

$$K_{p}^{q}\left(u\right)=\ln_{q}\left[M_{p}^{q}\right].$$

Notice that $B_{p,\infty }\left(0,1\right)\subset D_{M_{p}^{q}}$, where $B_{p,\infty }\left(0,1\right)$ is the open unit ball in $L^{\infty }\left(p.\mu \right)$. Some properties of the functional $K_{p}^{q}$ are described in the theorem below.

**Theorem** **1** ([15], Theorem 9)**.** *The cumulant generating function $K_{p}^{q}$ satisfies:*

(1) The function $z=e_{q}^{u{⊖}_{q}K_{p}^{q}\left(u\right)}p$ is a probability density on $P_{\mu }$, since $u\in B_{p,\infty }\left(0,1\right)$;
(2) *$K_{p}^{q}$ is infinitely Fréchet differentiable and its n-th derivative evaluated at the directions $\left(v_{1},\ldots ,v_{n}\right)\in B_{p,\infty }\left(0,1\right)\times \ldots \times B_{p,\infty }\left(0,1\right)$, is of the form*
  $$D^{n}K_{p}^{q}\left(u\right).\left(v_{1}\ldots v_{n}\right)={\left[M_{p}^{q}\left(u\right)\right]}^{1-q}Q_{n}\left(q\right)\int_{T}\left(v_{1}\ldots v_{n}\right)d\mu ;$$
(3) The functional $K_{p}^{q}$ is analytic in $B_{p,\infty }\left(0,1\right)$.

The function $K_{p}^{q}$ is used to define the *q*-exponential models

$$e_{q,p}:V_{p}\to P_{\mu },$$

where

(3) $$e_{q,p}\left(u\right)=e_{q}^{\left(u{⊖}_{q}K_{p}^{q}\left(u\right)\right)}p.$$

Moreover, the set

(4) $$B_{p}=\left\{u\in L^{\infty }\left(p.\mu \right);\int_{T}upd\mu =0\right\}$$

is a Banach space and

(5) $$V_{p}=\{u\in B_{p}{;||u||}_{p,\infty }<1\}$$

is the open unit ball of $B_{p}$. Since ${\left||u|\right|}_{p,\infty }<1,$ we obtain $\frac{-1}{1-q}<u$. Therefore $\frac{-1}{1-q}<\frac{u-K_{p}^{q}\left(u\right)}{1+\left(1-q\right)K_{p}^{q}\left(u\right)}=u{⊖}_{q}K_{p}^{q}\left(u\right)$ and consequently $e_{q,p}\left(u\right)=e_{q}^{\left(u{⊖}_{q}K_{p}^{q}\left(u\right)\right)}p$ is well defined.

The inverse of $e_{q,p}$ is given by [15]

$$e_{q,p}^{-1}\left(z\right)=\frac{\ln_{q}\left(\frac{z}{p}\right)-\int_{T}\ln_{q}\left(\frac{z}{p}\right)pd\mu }{1+\left(1-q\right)\int_{T}\ln_{q}\left(\frac{z}{p}\right)pd\mu }.$$

The transition map $e_{q,p_{2}}^{-1}\circ e_{q,p_{1}}:e_{q,p_{1}}^{-1}\left(U_{p_{1}}\cap U_{p_{2}}\right)\to e_{q,p_{2}}^{-1}\left(U_{p_{1}}\cap U_{p_{2}}\right)$, where $U_{p}$ is the range of $e_{q,p}$, is expressed as [15]

$$e_{q,p_{2}}^{-1}\left(e_{q,p_{1}}\left(u\right)\right)=\frac{u+\left[1+\left(1-q\right)u\right]\ln_{q}\left(\frac{p_{1}}{p_{2}}\right)-\int_{T}u+\left[1+\left(1-q\right)u\right]\ln_{q}\left(\frac{p_{1}}{p_{2}}\right)p_{2}d\mu }{1+\left(1-q\right)\int_{T}u+\left[1+\left(1-q\right)u\right]\ln_{q}\left(\frac{p_{1}}{p_{2}}\right)p_{2}d\mu },$$

where $p_{1},p_{2}\in P_{\mu }$ with $U_{p_{1}}\cap U_{p_{2}}\neq \emptyset$ and $u\in e_{q,p_{1}}^{-1}\left(U_{p_{1}}\cap U_{p_{2}}\right)$.

The map $e_{q,p}$ is injective and the set $e_{q,p}^{-1}\left(U_{p_{1}}\cap U_{p_{2}}\right)$ is open in the $B_{p_{1}}$-topology, where $p_{1},\;p_{2}\in P_{\mu }$. Hence, the transition map $e_{q,p_{2}}^{-1}\circ e_{q,p_{1}}$ is a topological homeomorphism and consequently the collection of pairs ${\left\{\left(U_{p},e_{q,p}^{-1}\right)\right\}}_{p\in P_{\mu }}$ is a $C^{\infty }$-atlas modeled on $B_{p}$. Then, $P_{\mu }$ is a $C^{\infty }$-Banach manifold, since $e_{q,p}$ is a parametrization.

There exists a relation between the constructed manifold and the Tsallis relative entropy. In fact, let us consider, for t≠0 and 0<q<1, the following function

$$f\left(t\right)=-t\ln_{q}\left(\frac{1}{t}\right),$$

where $\ln_{q}\left(x\right)=\frac{x^{1-q}-1}{1-q},\;\mathrm{if}\;x>0$. Given *p* and *z* in $P_{\mu }$, the Tsallis divergence, also called *q*-divergence of *z* with relation to *p*, is expressed by

(6) $$I^{\left(q\right)}\left(z||p\right)=\int_{T}pf\left(\frac{z}{p}\right)d\mu .$$

**Proposition** **1** ([15], Proposition 16)**.** *Taking p, z in $P_{\mu }$, we obtain*

(1) $I^{\left(q\right)}\left(z||p\right)\geq 0$, with equality iff p=z.
(2) $I^{\left(q\right)}\left(z||p\right)\leq \int_{T}\left(z-p\right)f^{\prime }\left(\frac{z}{p}\right)d\mu$.

### 2.2. Musielak–Orlicz Spaces and φ-Families of Probability Distributions

Consider (T,Σ,μ) a σ-finite, non-atomic measure space. Let $P_{\mu }=\left\{p\in L^{0};p>0\;\mathrm{and}\;\int_{T}pd\mu =1\right\}$, where $L^{0}$ is the linear space of all real-valued, measurable functions on *T*, with equality μ-a.e. t∈T. The map Φ:T×[0,∞)→[0,∞] is a Musielak–Orlicz function if, for μ-a.e. (almost everywhere) t∈T, the following conditions hold [17]:

(1) Φ(t,·) is convex and lower semi-continuous;
(2) $\Phi \left(t,0\right)=\lim_{u↓0}\Phi \left(t,u\right)=0$ and Φ(t,∞)=∞;
(3) Φ(·,u) is measurable for each u≥0.

Since the items (1) and (2) occur, it follows that Φ(t,.) is not equal to 0 or *∞* in the interval (0,∞).

Consider the functional $I_{\Phi }\left(u\right)=\int_{T}\Phi \left(t,|u\left(t\right)|\right)d\mu$, for any $u\in L^{0}$. The Musielak–Orlicz space, Musielak–Orlicz class, Morse–Transue space associated the a Musielak–Orlicz function Φ are defined, respectively, by

$$L^{\Phi }=\left\{u\in L^{0};\;I_{\Phi }\left(\lambda u\right)<\infty \;\mathrm{for}\;\mathrm{each}\;\lambda \in \left(-\varepsilon ,\varepsilon \right),\;\mathrm{there}\;\mathrm{exists}\;\varepsilon >0\right\},$$

$${\tilde{L}}^{\Phi }=\left\{u\in L^{0};\;I_{\Phi }\left(u\right)<\infty \right\}$$

and

$$E^{\Phi }=\left\{u\in L^{0};I_{\Phi }\left(\lambda u\right)<\infty \;\mathrm{for}\;\mathrm{all}\;\lambda >0\right\}.$$

Consider the Luxemburg norm

$${∥u∥}_{\Phi }=\inf\;\left\{\lambda >0;\;I_{\Phi }\left(\frac{u}{\lambda }\right)\leq 1\right\},$$

and the Orlicz norm

$${∥u∥}_{\Phi ,0}=\sup\left\{\left|\int_{T}uvd\mu \right|\;;\;v\in {\tilde{L}}^{\Phi^{*}}\;\mathrm{and}\;I_{\Phi^{*}}\left(v\right)\leq 1\right\},$$

where $\Phi^{*}\left(t,v\right)=\sup_{u\geq 0}\left(uv-\Phi \left(t,u\right)\right)$ is the Fenchel conjugate of Φ(t,·). The Musielak–Orlicz space $L^{\Phi }$ equipped with one of these two norms is a Banach space. The norms above are equivalent and the inequalities ${∥u∥}_{\Phi }\leq {∥u∥}_{\Phi ,0}\leq 2{∥u∥}_{\Phi }$ hold for all $u\in L^{\Phi }$. For more details see [18,19].

Define the Musielak–Orlicz function as

(7) $$\Phi_{c}\left(t,u\right)=\varphi \left(t,c\left(t\right)+u\right)-\varphi \left(t,c\left(t\right)\right),$$

where c:T→R is a measurable function such that φ(t,c(t)) is μ-integrable and we write $L_{c}^{\varphi }$, ${\tilde{L}}_{c}^{\varphi }$ and $E_{c}^{\varphi }$, in the place of $L^{\Phi_{c}}$, ${\tilde{L}}^{\Phi_{c}}$ and $E^{\Phi_{c}}$ respectively. In [10] it was defined the parametrization

$$\varphi_{c}:B_{c}^{\varphi }\to F_{c}^{\varphi },$$

where

(8) $$\varphi_{c}\left(u\right)=\varphi \left(c+u-\psi \left(u\right)u_{0}\right),$$

for each $u\in B_{c}^{\varphi }=B_{c}^{\varphi }\cap K_{c}^{\varphi }$, and

(9) $$B_{c}^{\varphi }=\left\{u\in L_{c}^{\varphi };\int_{T}u\varphi_{+}^{\prime }\left(c\right)d\mu =0\right\},$$

(10) $$K_{c}^{\varphi }=\left\{u\in L_{c}^{\varphi };\;\int_{T}\;\varphi \left(c+\lambda u\right)<\infty \;\mathrm{for}\;\mathrm{each}\;\lambda \in \left(-\varepsilon ,1+\varepsilon \right),\;\mathrm{there}\;\mathrm{exists}\;\varepsilon >0\right\}.$$

The application $\psi :B_{c}^{\varphi }\to \left[0,\infty \right)$ is called the normalizing function and it is defined in such a way that $\varphi_{c}\left(u\right)=\varphi \left(c+u-\psi \left(u\right)u_{0}\right)$ is in $P_{\mu }$. We have that $⋃\left\{F_{c}^{\varphi };\varphi \left(c\right)\in P_{\mu }\right\}=P_{\mu }$, $\varphi_{c_{1}}^{-1}\left(F_{c_{1}}^{\varphi }\cap F_{c_{2}}^{\varphi }\right)$ and $\varphi_{c_{2}}^{-1}\left(F_{c_{1}}^{\varphi }\cap F_{c_{2}}^{\varphi }\right)$ are open for any $c_{1},c_{2}:T\to R$ measurable such that $\varphi (c_{1})$ and $\varphi (c_{2})$ are in $P_{\mu }$. The transition map is a $C^{\infty }$-isomorphism and consequently $\varphi_{c}$ is a parametrization.

In the next section, we will use the generalized open exponential arcs to build a parametrization to $P_{\mu }$.

## 3. Construction of Generalized φ-Families of Probability Distributions

Let (T,Σ,μ), be a σ-finite, non-atomic measure space and consider a deformed exponential function φ:T×R→[0,∞). In other words, φ(t,·) is convex for μ-a.e. t∈T and the limits $\lim_{u\to -\infty }\varphi \left(t,u\right)=0$, $\lim_{u\to \infty }\varphi \left(t,u\right)=\infty$ for μ-a.e. t∈T hold. In this work we consider two additional conditions on the deformed exponential φ:

(a1) φ(t,x)=0, for all $x<a_{\varphi },$ where $a_{\varphi }=\inf\left\{x\in R;\varphi (x)>0\right\};$
(a2) given a measurable function c:T→R such that $\int_{T}\varphi \left(t,c\left(t\right)\right)d\mu =1$, we have
  (11) $$\int_{T}\varphi \left(t,c\left(t\right)+\lambda \right)d\mu <\infty ,\;\mathrm{for}\;\mathrm{all}\;\lambda >0.$$

For a measurable function q:T→(0,1), we define the *q*-deformed exponential function $\exp_{q}:T\times R\to \left[0,\infty \right)$ as $\exp_{q}\left(t,u\right)=\exp_{q(t)}\left(u\right)$, where

$$\exp_{q}\left(u\right)={\left[1+\left(1-q\right)u\right]}_{+}^{1/(1-q)},$$

and ${\left[1+\left(1-q\right)u\right]}_{+}=\max\left\{1+\left(1-q\right)u,0\right\}$. In this case, the *q*-deformed exponential function satisfies the condition (a1) with $a_{\varphi }=\frac{-1}{1-q}$. In the next example, we prove that the *q*-deformed exponential function satisfies the condition (a2) for 0<q<1.

**Example** **1.** *Given α≥1, we consider two cases:*

*If u≤0, we have that αu≤u. Then,*

$$\begin{array}{ll} \exp_{q}\left(\alpha u\right) & \leq \exp_{q}\left(u\right)\\ & \leq \alpha^{\frac{1}{1-q}}\exp_{q}\left(u\right). \end{array}$$

*If u>0, we obtain*

$$\begin{array}{ll} \exp_{q}\left(\alpha u\right) & ={\left(1+(1-q)\alpha u\right)}^{\frac{1}{1-q}}\\ & ={\left(\alpha \alpha^{-1}+\left(1-q\right)\alpha u\right)}^{\frac{1}{1-q}}\\ & =\alpha^{\frac{1}{1-q}}\left(\alpha^{-1}+\left(1-q\right)u\right){)}^{\frac{1}{1-q}}\\ & \leq \alpha^{\frac{1}{1-q}}{\left(1+\left(1-q\right)u\right))}^{\frac{1}{1-q}}\\ & =\alpha^{\frac{1}{1-q}}\exp_{q}\left(u\right). \end{array}$$

*By the convexity property of $\exp_{q}\left(t,.\right)$, we obtain for any λ∈(0,1) that*

$$\begin{array}{ll} \exp_{q}\left(c+u\right) & \leq \lambda \exp_{q}\left(\lambda^{-1}c\right)+\left(1-\lambda \right)\exp_{q}\left({\left(1-\lambda \right)}^{-1}u\right)\\ & \leq \lambda^{1-1/(1-q)}\exp_{q}\left(c\right)+{\left(1-\lambda \right)}^{1-1/(1-q)}\exp_{q}\left(u\right). \end{array}$$

*Then, any positive function $u_{0}:T\to \left(0,\infty \right)$ such that $\int_{T}\exp_{q}\left(u_{0}\right)d\mu <\infty$ satisfies $\int_{T}\exp_{q}\left(c+\lambda u_{0}\right)d\mu <\infty$ for all λ>0.*

Now, we provide an example of a deformed exponential function that satisfies condition (a1), but does not satisfy condition (a2).

**Example** **2.** *Consider the function*

$$\varphi \left(u\right)=\left\{\begin{array}{cc} e^{{\left(u+1\right)}^{2}/2}, & u\geq 0\\ e^{1/2}\left(u+1\right), & -1\leq u\leq 0,\\ 0, & u\leq -1 \end{array}\right.$$

*where the measure μ is σ-finite and non atomic. Note that φ is convex, and satisfies φ(x)=0, for all $x<a_{\varphi },$ where $a_{\varphi }=\inf\left\{x\in R;\varphi \left(x\right)>0\right\}$ and $\lim_{u\to \infty }\varphi \left(u\right)=\infty .$ We will find a measurable function c:T→R with $\int_{T}\varphi \left(c\right)d\mu <\infty ,$ but $\int_{T}\varphi \left(c+\lambda \right)d\mu =\infty ,$ for some λ>0. For each m≥1, we consider*

$$v_{m}\left(t\right):=\left(m\log\left(2\right)-\frac{3}{2}\right)1_{E_{m}}\left(t\right),$$

*where $E_{m}=\left\{t\in T;\;m\log\left(2\right)-\frac{3}{2}>0\right\}$ and $1_{E_{m}}\left(t\right)=\left\{\begin{array}{cc} 1, & t\in E_{m}\left(t\right)\\ 0, & t\notin E_{m}\left(t\right) \end{array}\right.$. Since $v_{m}↑\infty ,$ we can find a subsequence $\left\{v_{m_{n}}\right\}$ such that*

$$\int_{E_{m_{n}}}e^{{\left(v_{m_{n}}+2\right)}^{2}/2}d\mu \geq 2^{n}.$$

*According to [17], there exists a subsequence $w_{k}=v_{m_{n_{k}}}$ and pairwise disjoint sets $A_{k}\subseteq E_{m_{n_{k}}}$ for which*

$$\int_{A_{k}}e^{{\left(v_{m_{n}}+2\right)}^{2}/2}d\mu =1.$$

*Let us define $c=\bar{c}1_{T\setminus A}+\sum_{k=1}^{\infty }w_{k}1_{A_{k}}$ where $A={⋃}_{k=1}^{\infty }A_{k}$ and $\bar{c}$ is any measurable function such that $\varphi (\bar{c}\left(t\right))>0$ for t∈T∖A and $\int_{T\setminus A}\varphi \left(\bar{c}\right)d\mu <\infty .$ Observing that*

$$e^{{\left(w_{k}\left(t\right)+2\right)}^{2}/2}=2^{m_{n_{k}}}e^{{\left(w_{k}\left(t\right)+1\right)}^{2}/2},\;\;for\;t\in A_{k},$$

*we obtain*

$$\int_{A_{k}}e^{({w_{k}\left(t\right)+1)}^{2}/2}d\mu =\frac{1}{2^{m_{n_{k}}}},\;\;for\;every\;m\geq 1.$$

*Hence, we can write*

$$\begin{array}{cc} \int_{T}\varphi \left(c\right)d\mu = & \int_{T\setminus A}\varphi \left(\bar{c}\right)d\mu +\sum_{k=1}^{\infty }\int_{A_{k}}e^{({w_{k}\left(t\right)+1)}^{2}/2}d\mu \\ = & \int_{T\setminus A}\varphi \left(\bar{c}\right)d\mu +\sum_{k=1}^{\infty }\frac{1}{2^{m_{n_{k}}}}<\infty . \end{array}$$

*On the other hand, we also have*

$$\begin{array}{cc} \int_{T}\varphi \left(c+1\right)d\mu = & \int_{T\setminus A}\varphi \left(\bar{c}\right)d\mu +\sum_{k=1}^{\infty }\int_{A_{k}}e^{({w_{k}\left(t\right)+2)}^{2}/2}d\mu \\ = & \int_{T\setminus A}\varphi \left(\bar{c}\right)d\mu +\sum_{k=1}^{\infty }1\\ = & \infty , \end{array}$$

*which shows that (a2) is not satisfied.*

**Definition** **2.** *We say that p and z in $P_{\mu }$ are φ-connected by an open arc, if there exists an open interval I⊃[0,1] and a constant κ(α), such that*

(12) $$\varphi \left(\left(1-\alpha \right)\varphi^{-1}\left(p\right)+\alpha \varphi^{-1}\left(z\right)-\kappa \left(\alpha \right)\right)\;\in P_{\mu },$$

*for each α∈I, where κ(α) depends of α, p and z.*

According to the proof proved in [11], we have that κ(α)≤0 for each α∈[0,1]. Indeed,

- for α=0,1, we have clearly that κ(α)=0;
- for α∈(0,1), the convexity of the function of the φ ensures that $0\leq \varphi (\left(1-\alpha \right)\varphi^{-1}\left(p\right)+\alpha \varphi^{-1}\left(z\right))<\left(1-\alpha \right)p+\alpha z$. Integrating the inequality we obtain
  $$0\leq \int_{T}\varphi \left(\left(1-\alpha \right)\varphi^{-1}\left(p\right)+\alpha \varphi^{-1}\left(z\right)\right)d\mu \leq 1.$$
  Since κ(α) satisfies
  $$\int_{T}\varphi \left(\left(1-\alpha \right)\varphi^{-1}\left(p\right)+\alpha \varphi^{-1}\left(z\right)-\kappa \left(\alpha \right)\right)d\mu =1,$$
  then κ(α)≤0, for α∈[0,1].

Now we will define, by using generalized exponential arcs, important sets for the construction of generalized φ-family of probability distributions. Let us define

$$\tilde{\kappa }\left(\alpha \right)=\sup\left\{\lambda >0;\exists \;\varepsilon >0\;\mathrm{where}\;\left(1-\alpha \right)\varphi^{-1}\left(p\right)+\alpha \varphi^{-1}\left(z\right)-\lambda >a_{\varphi },\;\mu -a.e.\;t\in T,\;\mathrm{for}\;\mathrm{each}\;\alpha \in \left(-\varepsilon ,1+\varepsilon \right)\right\}.$$

as *p* and *z* are φ-connected by an open arc, we have that $\left(1-\alpha \right)\varphi^{-1}\left(p\right)+\alpha \varphi^{-1}\left(z\right)-\kappa \left(\alpha \right)>a_{\varphi }$, for each α∈I. Hence, $\kappa \left(\alpha \right)<\tilde{\kappa }\left(\alpha \right)$, i.e., $\kappa \left(\alpha \right)\in [-\infty ,\tilde{\kappa }\left(\alpha \right)).$ For $p\in P_{\mu }$, where p=φ(c), consider the set

$$R_{c}^{\varphi }=\left\{q\in P_{\mu };\exists \;\varepsilon >0\;\mathrm{where}\;\left(1-\alpha \right)\varphi^{-1}\left(p\right)+\alpha \varphi^{-1}\left(z\right)-\tilde{\kappa }\left(\alpha \right)\geq a_{\varphi },\;\mu -a.e.\;t\in T,\;\mathrm{for}\;\mathrm{each}\;\alpha \in \left(-\varepsilon ,1+\varepsilon \right)\right\}.$$

We will show that the set

(13) $$A_{c}^{\varphi }=\left\{q\in R_{c}^{\varphi };\exists \;\varepsilon >0\;\mathrm{where}\;\int_{T}\varphi \left(\left(1-\alpha \right)\varphi^{-1}\left(p\right)+\alpha \varphi^{-1}\left(z\right)-\tilde{\kappa }\left(\alpha \right)\right)d\mu <1,\mathrm{for}\;\mathrm{each}\alpha \in \left(-\varepsilon ,1+\varepsilon \right)\right\}$$

is a generalized φ-family of probability distributions.

Consider the partition of $P_{\mu }$ into equivalence classes using the following relation: given *p*, *z* ∈ $P_{\mu }$ we say that p∼z if and only if *p* and *z* are φ-connected by an open arc. This equivalence relation is necessary to define an atlas modeled on Banach spaces.

Consider then $L_{c}^{\varphi }$ be the Musielak–Orlicz space, given as

$$L_{c}^{\varphi }=\left\{u\in L^{0};\exists \;\varepsilon >0\;\mathrm{where}\;\int_{T}\varphi \left(c+\lambda u\right)d\mu <\infty ,\;\mathrm{for}\;\mathrm{each}\;\lambda \in \left(-\varepsilon ,\varepsilon \right)\right\}$$

and the set

$$N_{c}^{\varphi }=\left\{u\in L_{c}^{\varphi };\exists \;\varepsilon \in \left(0,1\right)\;\mathrm{where}\;\;c+\lambda u\geq a_{\varphi },\;\mathrm{for}\;\mathrm{each}\;\lambda \in \left[-\varepsilon ,\varepsilon \right],\right\}.$$

**Lemma** **1.** *The set* $N_{c}^{\varphi }$ *is a closed subspace.*

**Proof.**  Clearly $0\in N_{c}^{\varphi }.$ Given $u,v\in N_{c}^{\varphi },$ there exist $\varepsilon_{1,}\varepsilon_{2}\in \left(0,1\right),$ such that

$$c+\lambda u\geq a_{\varphi },\;\mu -a.e.,\;\mathrm{for}\;\mathrm{each}\;\lambda \in \left[-\varepsilon_{1,}\varepsilon_{1}\right]$$

and

$$c+\lambda v\geq a_{\varphi },\;\mu -a.e.,\;\mathrm{for}\;\mathrm{each}\;\lambda \in \left[-\varepsilon_{2,}\varepsilon_{2}\right].$$

Considering $\varepsilon =min\{\varepsilon_{1,}\varepsilon_{2}\},$ we have that $u+v\in N_{c}^{\varphi }$. Finally, given α∈R we obtain $\alpha u\in N_{c}^{\varphi }$, since $c+\lambda \left(\alpha u\right)\geq a_{\varphi },\;\mu -a.e.,\;\mathrm{for}\;\mathrm{each}\;\lambda \in \left[-\frac{\varepsilon_{1}}{\alpha },\frac{\varepsilon_{1}}{\alpha }\right]$.

The fact that remains to show is that $N_{c}^{\varphi }$ is closed. For this, let $\left(u_{n}\right)\in N_{c}^{\varphi },$ convergent μ-a.e. for $u\in L_{c}^{\varphi }$. This implies that there exists a subsequence $\left(u_{n}\right)$, such that $c+\lambda u_{n}\to c+\lambda u,\mu -$a.e. t∈T.

Then, for each n∈N we can find $\varepsilon_{n}\in \left(0,1\right),$ with $c+\lambda u_{n}\geq a_{\varphi },\mu -$a.e. t∈T, for each $\lambda \in [-\varepsilon_{n,}\varepsilon_{n}].$

The compactness of $\left[-\varepsilon_{n,}\varepsilon_{n}\right]$ ensures that the coverage $\left\{(-{\bar{\varepsilon }}_{n}-\delta ,{\bar{\varepsilon }}_{n}+\delta );n\in N\right\}$ admits a finite undercoverage. Let $\left\{-{\bar{\varepsilon }}_{1}-\delta ,\varepsilon_{1}+\delta ,\ldots ,-{\bar{\varepsilon }}_{n_{0}}-\delta ,\varepsilon_{n_{0}}+\delta \right\}$ the set of the elements that constitute the finite undercoverage. Taking $\bar{\varepsilon }=\min\left\{-{\bar{\varepsilon }}_{1}-\delta ,{\bar{\varepsilon }}_{1}+\delta ,\ldots ,-{\bar{\varepsilon }}_{n_{0}}-\delta ,{\bar{\varepsilon }}_{n_{0}}+\delta \right\},$ it follows that $c+\lambda u_{n}\geq a_{\varphi },$μ-a.e. t∈T, for each $\lambda \in [-{\bar{\varepsilon }}_{,}\bar{\varepsilon }].$

Passing to the limit, we obtain $c+\lambda u\geq a_{\varphi },\mu -$a.e. t∈T, for each $\lambda \in [-\bar{\varepsilon },\bar{\varepsilon }].$ Therefore, $u\in N_{c}^{\varphi }$ and consequently $N_{c}^{\varphi }$ is closed.  □

Define the set

(14) $${\tilde{K}}_{c}^{\varphi }=\left\{u\in N_{c}^{\varphi };\;\exists \;\varepsilon \in \left(0,1\right),\;\mathrm{such}\;\mathrm{that}\;\int_{T}\varphi \left(c+\lambda u\right)d\mu <\infty ,\;\mathrm{for}\;\mathrm{each}\;\lambda \in \left(-\varepsilon ,1+\varepsilon \right)\right\}.$$

**Lemma** **2.** *The set ${\tilde{K}}_{c}^{\varphi }$ is open in $N_{c}^{\varphi }$.*

**Proof.**  Let $u\in {\tilde{K}}_{c}^{\varphi }$. Then, there exists ε∈(0,1), such that $\int_{T}\varphi \left(c+\alpha u\right)d\mu <\infty$ for each α∈[−ε,1+ε] and $u\in N_{c}^{\varphi }$. Considering $\delta ={\left[\frac{2}{\varepsilon }\left(1+\varepsilon \right)\left(1+\frac{\varepsilon }{2}\right)\right]}^{-1}$, we have that for any $v\in B_{\delta }=\left\{w\in N_{c}^{\varphi }{;||w||}_{\Phi_{c}}<\delta \right\}$ it occurs $I_{\Phi_{c}}\left(\frac{v}{\delta }\right)\leq 1$ and consequently $\int_{T}\varphi \left(c+\frac{1}{\delta }\left|v\right|\right)d\mu \leq 2$. Given $\alpha \in \left(0,1+\frac{\varepsilon }{2}\right)$ we denote $\lambda =\frac{\alpha }{1+\varepsilon }$. The inequality

$$\frac{\alpha }{1-\lambda }=\frac{\alpha }{1-\frac{\alpha }{1+\varepsilon }}\leq \frac{1+\frac{\varepsilon }{2}}{1-\frac{1+\frac{\varepsilon }{2}}{1+\varepsilon }}=\frac{2}{\varepsilon }\left(1+\varepsilon \right)\left(1+\frac{\varepsilon }{2}\right)=\frac{1}{\delta },$$

implies

$$\begin{array}{ll} \varphi (c+\alpha (u+v)) & \leq \varphi \left(\lambda \varphi \left(c+\frac{\alpha }{\lambda }\right)+\left(1-\lambda \right)\varphi \left(c+\frac{\alpha }{1-\lambda }v\right)\right)\\ & \leq \lambda \varphi \left(c+\frac{\alpha }{\lambda }\right)+\left(1-\lambda \right)\varphi \left(c+\frac{\alpha }{1-\lambda }v\right)\\ & \leq \lambda \varphi \left(c+\left(1+\epsilon \right)u\right)+\left(1-\lambda \right)\varphi \left(c+\frac{1}{\delta }\left|v\right|\right). \end{array}$$

For $\alpha \in \left(-\frac{\varepsilon }{2},0\right),$ we can write

$$\begin{array}{ll} \varphi (c+\alpha (u+v)) & \leq \frac{1}{2}\varphi \left(c+2\alpha u\right)+\frac{1}{2}\varphi \left(c+2\alpha v\right)\\ & \leq \frac{1}{2}\varphi \left(c+2\alpha u\right)+\frac{1}{2}\varphi \left(c+|v|\right). \end{array}$$

Then, we have

$$\int_{T}\varphi \left(c+\alpha \left(u+v\right)\right)d\mu <\infty ,$$

for any $\alpha \in \left(-\frac{\varepsilon }{2},1+\frac{\varepsilon }{2}\right)$. Hence, $u+v\in K{}_{c}^{\varphi }$ and since $N_{c}^{\varphi }$ is a subspace, we obtain $u+v\in {\tilde{K}}_{c}^{\varphi }$. As a consequence, $B_{\delta }\left(u\right)$ is contained in ${\tilde{K}}_{c}^{\varphi }$ and therefore the set ${\tilde{K}}_{c}^{\varphi }$ is open.  □

The set ${\tilde{K}}_{c}^{\varphi }$ defined in (14) is important to guarantee that φ(c+αu) may be in $P_{\mu }$. Now, we establish a relationship between the connection by an open arc and ${\tilde{K}}_{c}^{\varphi }$ similar to that was proved in [14].

**Proposition** **2.** *Fix $p\in P_{\mu }$. We say that $z\in P_{\mu }$ is φ-connected to p by an open arc, if and only if, there exists an open interval I⊃[0,1] and a random variable $u\in L_{c}^{\varphi }$, such that $p\left(\alpha \right)\propto \varphi \left(c+\alpha u\right)\in P_{\mu }$, for each α∈I and p(0)=p and p(1)=z.*

**Proof.**  Since that *z* is φ-connected to p by an open arc, there exists an interval I⊃[0,1], such that $\int_{T}\varphi \left(\left(1-\alpha \right)\varphi^{-1}\left(p\right)+\alpha \varphi^{-1}\left(z\right)\right)d\mu <\infty$, for each α∈I. Considering $u=\varphi^{-1}\left(z\right)-\varphi^{-1}\left(p\right)$, we have

$$\begin{array}{ll} \int_{T}\varphi \left(c+\alpha u\right)d\mu & =\int_{T}\varphi \left(\varphi^{-1}\left(p\right)+\alpha \left(\varphi^{-1}\left(z\right)-\varphi^{-1}\left(p\right)\right)\right)d\mu \\ & =\int_{T}\varphi \left(\left(1-\alpha \right)\varphi^{-1}\left(p\right)+\alpha \varphi^{-1}\left(z\right)\right)d\mu , \end{array}$$

where $u=\varphi^{-1}\left(z\right)-\varphi^{-1}\left(p\right)$ and φ(c)=p. Therefore $u\in L_{c}^{\varphi }$. Another conclusion that arises from the fact of *q* is φ-connected to *p* by a open arc is that $\left(1-\alpha \right)\varphi^{-1}\left(p\right)+\alpha \varphi^{-1}\left(z\right)-\kappa \left(\alpha \right)>a_{\varphi }$. Hence, $p\left(\alpha \right)\propto \varphi \left(c+\alpha u\right)\in P_{\mu }$, for each α∈I and p(0)=p and p(1)=z.

Reciprocally, taking p(1)=q, we get φ(c+u)=z, and consequently $u=\varphi^{-1}\left(z\right)-\varphi^{-1}\left(p\right)$ with φ(c)=p=p(0).  □

One should notice that as a consequence of Proposition 2, given $p,\;z\in P_{\mu }$φ-connected by an open arc, the random variable $u\in {\tilde{K}}_{c}^{\varphi }=K_{c}^{\varphi }\cap N_{c}^{\varphi }$. In fact, this follows from two reasons: as $p,\;z\in P_{\mu }$ it follows that $\varphi^{-1}\left(p\right),\;\varphi^{-1}\left(z\right)>a_{\varphi }$ and as *z* is φ-connected the *p* by an open arc we have $\int_{T}\varphi \left(c+\alpha u\right)d\mu <\infty$ for each α∈(−ε,1+ε).

**Remark** **1.** *Since the function φ-arc is injective, in the Proposition 2 only the case z≠p is considered. Therefore, there exists $z\in A_{c}^{\varphi }$ such that z≠p.*

**Lemma** **3.** *Let $z\in A_{c}^{\varphi }$φ-connected to p by an open arc. The map*

$$V\left(\lambda \right)=\int_{T}\varphi \left(\left(1-\alpha \right)\varphi^{-1}\left(p\right)+\alpha \varphi^{-1}\left(z\right)-\lambda \right)d\mu$$

*is then well defined. Moreover,* V(λ) *is strictly increasing.*

**Proof.**  Proposition 2 ensures that $p\left(\alpha \right)\propto \varphi \left(c+\alpha u\right)\in P_{\mu }$, where $u=\varphi^{-1}\left(z\right)-\varphi^{-1}\left(p\right)\in {\tilde{K}}_{c}^{\varphi }$ and φ(c+u)=z. Then, we can find ε>0 such that $\varphi (c+\left(1+\varepsilon \right)\left(\varphi^{-1}\left(z\right)-\varphi^{-1}\left(p\right)\right))$ is μ-integrable. Given α∈(−ε,1+ε), taking $\bar{\lambda }=\frac{\alpha }{1+\varepsilon }$, we obtain

$$\begin{array}{ll} \varphi (\left(1-\alpha \right)\varphi^{-1}\left(p\right)+\alpha \varphi^{-1}\left(z\right)-\lambda ) & =\varphi \left(\bar{\lambda }\left(c+\frac{\alpha }{\bar{\lambda }}\left(\varphi^{-1}\left(z\right)-\varphi^{-1}\left(p\right)\right)\right)+\left(1-\bar{\lambda }\right)\left(c+\frac{\alpha }{1-\bar{\lambda }}\lambda \right)\right)\\ & \leq \bar{\lambda }\varphi \left(c+\frac{\alpha }{\bar{\lambda }}\left(\varphi^{-1}\left(z\right)-\varphi^{-1}\left(p\right)\right)\right)+\left(1-\bar{\lambda }\right)\varphi \left(c+\frac{\alpha }{1-\bar{\lambda }}\lambda \right), \end{array}$$

and consequently $\varphi (\left(1-\alpha \right)\varphi^{-1}\left(p\right)+\alpha \varphi^{-1}\left(z\right)-\lambda )$ is μ-integrable, for every λ∈R and for each α∈(−ε,1+ε). This proves that V(λ) is well defined. By the dominated convergence theorem, the map $\lambda \mapsto V\left(\lambda \right)=\int_{T}\varphi \left(\left(1-\alpha \right)\varphi^{-1}\left(p\right)+\alpha \varphi^{-1}\left(z\right)-\lambda \right)d\mu$ is continuous, $\lim_{\lambda \to \infty }V\left(\lambda \right)=0$ and $\lim_{\lambda \to -\infty }V\left(\lambda \right)=\infty$. Hence, given $\lambda \in \{\lambda \in R;\left(1-\alpha \right)\varphi^{-1}\left(p\right)+\alpha \varphi^{-1}\left(z\right)-\lambda >a_{\varphi },\;\mu -a.e.\;t\in T,$ foreachα∈(−ε,1+ε)}, we have that V(λ) is strictly increasing.  □

**Proposition** **3.** *Fix $p=\varphi \left(c\right)\in P_{\mu }$ and $z\in R_{c}^{\varphi }$. Then, $z\in A_{c}^{\varphi }$ if, and only if z is φ-connected the p by a open arc.*

**Proof.**  Given $z\in A_{c}^{\varphi }$ there exists ε>0, such that $\int_{T}\varphi \left(\left(1-\alpha \right)\varphi^{-1}\left(p\right)+\alpha \varphi^{-1}\left(z\right)-\tilde{\kappa }\left(\alpha \right)\right)d\mu <1$ and $\left(1-\alpha \right)\varphi^{-1}\left(p\right)+\alpha \varphi^{-1}\left(z\right)-\tilde{\kappa }\left(\alpha \right)\geq a_{\varphi }$, μ-a.e. t∈T for each α∈(−ε,1+ε). Then, $\int_{T}\varphi \left(\left(1-\alpha \right)\varphi^{-1}\left(p\right)+\alpha \varphi^{-1}\left(z\right)\right)d\mu <\infty$ for each α∈(−ε,1+ε) which ensures that *q* is φ-connected to *p* by an open arc.

Reciprocally, take $z\in R_{c}^{\varphi }$φ-connected to *p* by an open arc. In this way there exists ε>0, where $\int_{T}\varphi \left(\left(1-\alpha \right)\varphi^{-1}\left(p\right)+\alpha \varphi^{-1}\left(z\right)-\kappa \left(\alpha \right)\right)d\mu =1$ and $\left(1-\alpha \right)\varphi^{-1}\left(p\right)+\alpha \varphi^{-1}\left(z\right)-\kappa \left(\alpha \right)>a_{\varphi }$, for each α∈(−ε,1+ε). Note that

(15) $$\begin{array}{ll} \int_{T}\varphi \left(\left(1-\alpha \right)\varphi^{-1}\left(p\right)+\alpha \varphi^{-1}\left(z\right)-\tilde{\kappa }\left(\alpha \right)\right)d\mu & \leq \int_{T}\varphi \left(\left(1-\alpha \right)\varphi^{-1}\left(p\right)+\alpha \varphi^{-1}\left(z\right)-\kappa \left(\alpha \right)\right)d\mu \\ & =1, \end{array}$$

because $\kappa \left(\alpha \right)<\tilde{\kappa }\left(\alpha \right)$, for each α∈(−ε,1+ε) and φ is non-decreasing. Suppose that $z\notin A_{c}^{\varphi }$, there exists α∈(−ε,1+ε) such that

(16) $$\int_{T}\varphi \left(\left(1-\alpha \right)\varphi^{-1}\left(p\right)+\alpha \varphi^{-1}\left(z\right)-\tilde{\kappa }\left(\alpha \right)\right)d\mu \geq 1.$$

The Equations (15) and (16) ensure that $\int_{T}\varphi \left(\left(1-\alpha \right)\varphi^{-1}\left(p\right)+\alpha \varphi^{-1}\left(z\right)-\tilde{\kappa }\left(\alpha \right)\right)d\mu =1$ for each α∈(−ε,1+ε). Therefore, by Lemma 3 it exists a unique $\lambda_{0}$ satisfying $\left(1-\alpha \right)\varphi^{-1}\left(p\right)+\alpha \varphi^{-1}\left(z\right)-\lambda_{0}>a_{\varphi },$μ-a.e.t∈T, such that $V(\lambda_{0})=1$. Since κ(α) is such that $\left(1-\alpha \right)\varphi^{-1}\left(p\right)+\alpha \varphi^{-1}\left(z\right)-\kappa \left(\alpha \right)>a_{\varphi },\;\mu -a.e.\;t\in T,\;\mathrm{for}\;\mathrm{each}\;\alpha \in \left(-\varepsilon ,1+\varepsilon \right)$, it follows that $\lambda_{0}=\kappa \left(\alpha \right)$ and consequently κ(α) is unique. Hence, $\kappa \left(\alpha \right)=\tilde{\kappa }\left(\alpha \right)$ for each α∈(−ε,1+ε), that is an absurd.  □

By Corollary 3 the sets $A_{c}^{\varphi }$ are the connected components of $P_{\mu }$. Then, we need to find a domain for the parametrization in such a way that the image is $A_{c}^{\varphi }$.

We will make some similar considerations to the ones present in [10].

Remark that, for $u\in {\tilde{K}}_{c}^{\varphi }$, φ(c+u) is not necessarily in $P_{\mu }$. Define $\psi :{\tilde{K}}_{c}^{\varphi }\to R$, such that the density

(17) $$\varphi_{c}\left(u\right)=\varphi \left(c+u-\psi \left(u\right)\right)$$

is contained in $P_{\mu }$. We have that the open domain maximal of ψ is contained in ${\tilde{K}}_{c}^{\varphi }$. Note that ψ is well defined, since $c+u-\psi \left(u\right)>a_{\varphi },\;\mu -a.e.\;t\in T$. It can be then proved that $\psi :{\tilde{K}}_{c}^{\varphi }\to R$ is convex, and as a consequence $\psi :{\tilde{K}}_{c}^{\varphi }\to R$ is continuous, since ${\tilde{K}}_{c}^{\varphi }$ is open by Lemma 2.

Let $\varphi_{+}^{\prime }$ be the operator acting on the set of real-valued functions u:T→R given by $\varphi_{+}^{\prime }\left(u\right)\left(t\right)=\varphi_{+}^{\prime }\left(t,u\left(t\right)\right)$, where $\varphi_{+}^{\prime }\left(t,.\right)$ is the right-derivative of φ(t,.). Also, notice that the function $\psi :{\tilde{K}}_{c}^{\varphi }\to R$ can assume both positive and negative values. Consider the closed subspace

$${\tilde{B}}_{c}^{\varphi }=\left\{u\in N_{c}^{\varphi };\int_{T}u\varphi_{+}^{\prime }\left(c\right)d\mu =0\right\}.$$

Observe that the image of ψ will be contained in [0,∞), since the domain of ψ is restricted to a ${\tilde{B}}_{c}^{\varphi }$. By the convexity property of φ(t,.), we have

$$u\varphi_{+}^{\prime }\left(t,c\left(t\right)\right)\leq \varphi \left(t,c\left(t\right)+u\right)-\varphi \left(t,c\left(t\right)\right)\;\mathrm{for}\;\mathrm{all}\;u\in R.$$

Hence, we have that

$$1=\int u\varphi_{+}^{\prime }\left(c\right)d\mu +\int \varphi \left(c\right)d\mu \leq \int \varphi \left(c+u\right)d\mu <\infty \;\mathrm{for}\;\mathrm{any}\;u\in {\tilde{K}}_{c}^{\varphi }\cap {\tilde{B}}_{c}^{\varphi }={\tilde{B}}_{c}^{\varphi }.$$

Thus, it follows that ψ(u)≥0 in order to φ(c+u−ψ(u)) be in $P_{\mu }$.

Given a measurable function c:T→R such that p=φ(c) is a probability density in $P_{\mu }$. Consider the set

$$M_{c}^{\varphi }={\left(M_{c}^{\varphi }\right)}_{1}\cap {\left(M_{c}^{\varphi }\right)}_{2},$$

where

$${\left(M_{c}^{\varphi }\right)}_{1}=\left\{u\;\in \;{\tilde{B}}_{c}^{\varphi };\;c+\alpha \left(u-\psi \left(u\right)\right)-\tilde{\kappa }\left(\alpha \right)\right)>a_{\varphi }\;\mu -a.e.\;\mathrm{for}\;\mathrm{each}\;\alpha \in I⊃\left[0,1\right]\}$$

and

$${\left(M_{c}^{\varphi }\right)}_{2}=\left\{u\;\in \;{\tilde{B}}_{c}^{\varphi };\int_{T}\varphi \left(c+\alpha \left(u-\psi \left(u\right)\right)-\tilde{\kappa }\left(\alpha \right)\right))d\mu <1,\;\mathrm{for}\;\mathrm{each}\;\alpha \in I⊃\left[0,1\right]\right\}.$$

**Proposition** **4.** *Given $u\in M_{c}^{\varphi }$, we have that $\varphi \left(c+u-\psi \left(u\right)\right)\in A_{c}^{\varphi }$.*

**Proof.**  Given $u\in M_{c}^{\varphi }$, we have

$$c+\alpha \left(u-\psi \left(u\right)\right)+\tilde{\kappa }\left(\alpha \right))>a_{\varphi }$$

and

$$\int_{T}\varphi \left(c+\alpha u-\left(\alpha \psi \left(u\right)+\tilde{\kappa }\left(\alpha \right)\right)\right)d\mu <1,\;\mu -a.e.\;t\in T,\;\mathrm{for}\;\mathrm{each}\;\alpha \in I⊃\left[0,1\right].$$

Hence,

$$\left(1-\alpha \right)\varphi^{-1}\left(p\right)+\alpha \varphi^{-1}\left(\varphi \left(c+u-\psi \left(u\right)\right)\right)-\tilde{\kappa }\left(\alpha \right)=\left(1-\alpha \right)c+\alpha \left(c+u-\psi \left(u\right)\right)-\tilde{\kappa }\left(\alpha \right)=c+\alpha \left(u-\psi \left(u\right)\right)-\tilde{\kappa }\left(\alpha \right)>a_{\varphi },$$

for each α∈I⊃[0,1], which implies in $\varphi \left(c+u-\psi \left(u\right)\right)\in R_{c}^{\varphi }$. In addition,

$$\int_{T}\varphi (\left(1-\alpha \right)\varphi^{-1}\left(p\right)+\alpha \varphi^{-1}\left(\varphi \left(c+u-\psi \left(u\right)\right)-\tilde{\kappa }\left(\alpha \right)\right)d\mu =\int_{T}\varphi \left(c+\alpha \left(u-\psi \left(u\right)\right)-\tilde{\kappa }\left(\alpha \right)\right)d\mu <1,$$

for each α∈I⊃[0,1] and therefore, $\varphi \left(c+u-\psi \left(u\right)\right)\in A_{c}^{\varphi }$.  □

**Proposition** **5.** *The set $M_{c}^{\varphi }$ is open in $B_{c}^{\varphi }$.*

**Proof.**  Consider the sets

$${\left(M_{c}^{\varphi }\right)}_{1}=\left\{u\;\in \;{\tilde{B}}_{c}^{\varphi };\;c+\alpha \left(u-\psi \left(u\right)\right)-\tilde{\kappa }\left(\alpha \right)>a_{\varphi }\;\mu -a.e.\;\mathrm{for}\;\mathrm{each}\;\alpha \in I⊃\left[0,1\right]\right\}$$

and

$${\left(M_{c}^{\varphi }\right)}_{2}=\left\{u\;\in \;{\tilde{B}}_{c}^{\varphi };\int_{T}\varphi \left(c+\alpha \left(u-\psi \left(u\right)\right)-\tilde{\kappa }\left(\alpha \right)\right)d\mu <1,\;\mathrm{for}\;\mathrm{each}\;\alpha \in I⊃\left[0,1\right]\right\}.$$

Define the functions

$$f\left(\alpha ,u\right)=c+\alpha u-\alpha \psi \left(u\right)-\tilde{\kappa }\left(\alpha \right)\;and\;g\left(\alpha ,u\right)=\int_{T}\varphi \left(c+\alpha u-\alpha \psi \left(u\right)-\tilde{\kappa }\left(\alpha \right)\right)d\mu .$$

1. The function *f* is well defined and continuous, since $\psi :{\tilde{K}}_{c}^{\varphi }\to R$ is continuous;
2. The map *g* is well defined in ${\left(M_{c}^{\varphi }\right)}_{2}$ and continuous, since φ and ψ are continuous.

Moreover, given $u\in M_{c}^{\varphi }$, in particular $u\in {\left(M_{c}^{\varphi }\right)}_{1}$ and $u\in {\left(M_{c}^{\varphi }\right)}_{2}$. By the continuity of *f* and *g* respectively, exist $\varepsilon_{1},\;\varepsilon_{2}\in \left(0,1\right)$, such that for each $v_{1}\in B_{\varepsilon_{1}}\left(u\right)\subset B_{c}^{\varphi }$, we have $f\left(v_{1}\right)>a_{\varphi }$ and for each $v_{2}\in B_{\varepsilon_{2}}\left(u\right)\subset B_{c}^{\varphi }$, we have $g(v_{2})<1$. Taking, $\varepsilon =\min\left\{\varepsilon_{1},\varepsilon_{2}\right\}$, we obtain that $B_{\varepsilon }\left(u\right)\subset M_{c}^{\varphi }$ and consequently $M_{c}^{\varphi }$ is open in $B_{c}^{\varphi }$.  □

Clearly $P_{\mu }=⋃\left\{A_{c}^{\varphi };\varphi \left(c\right)\in P_{\mu }\right\}$. Consider the measurable functions $c_{1},c_{2}:T\to R$, where $p_{1}=\varphi \left(c_{1}\right)$ and $p_{2}=\varphi \left(c_{2}\right)$ belong to $P_{\mu }$. The parametrization $\varphi_{c_{1}}:M_{c_{1}}^{\varphi }\to A_{c_{1}}^{\varphi }$ and $\varphi_{c_{2}}:M_{c_{2}}^{\varphi }\to A_{c_{2}}^{\varphi }$ have a transition map given as

$$\varphi_{c_{2}}^{-1}\circ \varphi_{c_{1}}:\varphi_{c_{1}}^{-1}\left(A_{c_{1}}^{\varphi }\cap A_{c_{2}}^{\varphi }\right)\to \varphi_{c_{2}}^{-1}\left(A_{c_{1}}^{\varphi }\cap A_{c_{2}}^{\varphi }\right).$$

Given $\psi_{1}:M_{c_{1}}^{\varphi }\to \left[0,\infty \right)$ and $\psi_{2}:M_{c_{2}}^{\varphi }\to \left[0,\infty \right)$ being the normalizing functions associated to $c_{1}$ and $c_{2},$ respectively, and the functions $u\in M_{c_{1}}^{\varphi }$ and $v\in M_{c_{2}}^{\varphi }$ are such that $\varphi_{c_{1}}\left(u\right)=\varphi_{c_{2}}\left(v\right)\in A_{c_{1}}^{\varphi }\cap A_{c_{2}}^{\varphi }.$ So, we have

(18) $$v=c_{1}-c_{2}+u-\psi_{1}\left(u\right)+\psi_{2}\left(v\right).$$

Multiplying the Equation (18) by ${\left(\varphi \right)}_{+}^{\prime }\left(c_{2}\right)$ and integrating with respect to the measure μ, once the function *v* is in $M_{c_{2}}^{\varphi }$, we obtain

$$0=\int_{T}\left(c_{1}-c_{2}+u\right){\left(\varphi \right)}_{+}^{\prime }\left(c_{2}\right)d\mu -\psi_{1}\left(u\right)\int_{T}{\left(\varphi \right)}_{+}^{\prime }\left(c_{2}\right)d\mu +\psi_{2}\left(v\right)\int_{T}{\left(\varphi \right)}_{+}^{\prime }\left(c_{2}\right)d\mu ,$$

and we can write

$$\psi_{2}\left(v\right)=\frac{-\int_{T}\left(c_{1}-c_{2}+u\right){\left(\varphi \right)}_{+}^{\prime }\left(c_{2}\right)d\mu }{\int_{T}{\left(\varphi \right)}_{+}^{\prime }\left(c_{2}\right)d\mu }+\psi_{1}\left(u\right)\frac{\int_{T}{\left(\varphi \right)}_{+}^{\prime }\left(c_{2}\right)d\mu }{\int_{T}{\left(\varphi \right)}_{+}^{\prime }\left(c_{2}\right)d\mu }.$$

Therefore

$$v=c_{1}-c_{2}+u-\psi_{1}\left(u\right)-\frac{\int_{T}\left(c_{1}-c_{2}+u\right){\left(\varphi \right)}_{+}^{\prime }\left(c_{2}\right)d\mu }{\int_{T}{\left(\varphi \right)}_{+}^{\prime }\left(c_{2}\right)d\mu }+\psi_{1}\left(u\right)\frac{\int_{T}{\left(\varphi_{q}\right)}_{+}^{\prime }\left(c_{2}\right)d\mu }{\int_{T}{\left(\varphi_{q}\right)}_{+}^{\prime }\left(c_{2}\right)d\mu }.$$

Hence, the transition map $\varphi_{c_{2}}^{-1}\circ \varphi_{c_{1}}$ can be expressed as

(19) $$\varphi_{c_{2}}^{-1}\circ \varphi_{c_{1}}\left(w\right)=c_{1}-c_{2}+w-\frac{\int_{T}\left(c_{1}-c_{2}+w\right){\left(\varphi \right)}_{+}^{\prime }\left(c_{2}\right)d\mu }{\int_{T}{\left(\varphi \right)}_{+}^{{}^{\prime }}\left(c_{2}\right)d\mu },$$

for every $w\in \varphi_{c_{1}}^{-1}\left(A_{c_{1}}^{\varphi }\cap A_{c_{2}}^{\varphi }\right).$ Showing that *w* and $c_{1}-c_{2}$ are in $L_{c_{2}}^{\varphi }$ and the spaces $L_{c_{1}}^{\varphi }$ and $L_{c_{2}}^{\varphi }$ have equivalent norms we obtain that this transition map will be of class $C^{\infty }$.

In the next corollary we have that Musielak–Orlicz spaces are equal. The proof follows as the one provided in [14].

**Corollary** **1.** *Let $p,z\in P_{\mu }$φ-connected by an open arc, where p=φ(c) and $z=\varphi (\tilde{c})$. Then, $L_{c}^{\varphi }=L_{\tilde{c}}^{\varphi }$.*

**Proof.**  We have that *z* is φ-connected to *p* by a open arc. Then, by Corollary 3, we have that $\tilde{c}=c+u-\psi \left(u\right)$. The result follows immediately from [10].  □

It follows from Corollary 1 that $\varphi_{c_{2}}^{-1}\circ \varphi_{c_{1}}$ is of class $C^{\infty }$, and consequently, the set $\varphi_{c_{1}}^{-1}\left(A_{c_{1}}^{\varphi }\cap A_{c_{2}}^{\varphi }\right)$ is open in **$B_{c}^{\varphi }$.**

**Proposition** **6** ([14], Proposition 8)**.** *The relation given in the Definition 2 is an equivalence relation.*

**Proof.**  Since reflexivity and symmetry properties immediately follow from the definition, we will only prove transitivity. Let be $p,\;z,\;s\in P_{\mu }$, such that,

p(t)∝φ(c+tu),s(t)∝φ(c+tv),t∈(−ε,1+ε)

with p(0)=φ(c)=p, p(1)=φ(c+u)=z,s(0)=φ(c)=p, s(1)=φ(c+v)=s and $u,\;v\in N_{c}^{\varphi }$. Consider

z(t)∝φ(c+(1−t)u+tv)∝φ(c+u+t(v−u))

is defined with $c+u=\tilde{c},\;p\left(t\right)\propto \varphi \left(\tilde{c}+t\left(v-u\right)\right)$, where $z\left(0\right)=\varphi \left(\tilde{c}\right)=\varphi \left(c+u\right)=z$, $z\left(1\right)=\varphi (\tilde{c}+\left(v-u\right))=\varphi \left(c+v\right)=s$. Therefore *z* and *s* are φ-connected.  □

As a consequence of the Corollary 3 and of the Proposition 6 we have that the φ-families $A_{c}^{\varphi }$ are maximal, in the sense that $A_{c}^{\varphi }\cap A_{\tilde{c}}^{\varphi }=\emptyset$ or if $A_{c}^{\varphi }\cap A_{\tilde{c}}^{\varphi }\neq \emptyset$, then $A_{c}^{\varphi }=A_{\tilde{c}}^{\varphi }$.

Hence, we can write the following proposition.

**Proposition** **7.** *The collection ${\left\{\left(M_{c}^{\varphi },\varphi_{c}\right)\right\}}_{\varphi \left(c\right)\in P_{\mu }}$ equip $P_{\mu }$ with a $C^{\infty }$-differentiable structure.*

## 4. The Tangent Bundle

In the previous section, the expression of the transition application $\varphi_{c_{2}}^{-1}\circ \varphi_{c_{1}}$ was important to garantee that $P_{\mu }$ could be equipped with a $C^{\infty }$-Banach structure. Now, we will use the transition application to find the tangent space of $P_{\mu }$ at the point p=φ(c) and the tangent bundle.

Given $p\in P_{\mu }$, we consider the triple $(A_{c}^{\varphi };\varphi_{c}^{-1};v),$ where $A_{c}^{\varphi }$ is the φ-family, $\varphi_{c}$ is the parametrization and *v* is a vector in $\varphi_{c}^{-1}\left(A_{c}^{\varphi }\right)$ which is contained in the vector space $L^{\Phi_{c}}$.

Let us define the following equivalence relation:

$$\left(A_{c}^{\varphi };\varphi_{c}^{-1};v\right)∼\left(A_{\tilde{c}}^{\varphi };\varphi_{\tilde{c}}^{-1};w\right)\Leftrightarrow {\left(\varphi_{\tilde{c}}^{-1}\circ \varphi_{c}\right)}^{\prime }\left(\varphi_{c}\left(p\right)\right)\left(v\right)=w.$$

The class $\left[A_{c}^{\varphi };\varphi_{c}^{-1};v\right]$ is called the tangent vector of $P_{\mu }$ in *p* and the set of all classes is called the tangent space and is denoted by $T_{p}\left(P_{\mu }\right)$. For more details we refer the reader to [20].

The vector $v\in \varphi_{c}^{-1}\left(A_{c}^{\varphi }\right)$ is the velocity vector of a curve in the parametrization domain. In fact, consider $\left(A_{c_{1}}^{\varphi },\varphi_{c_{1}}^{-1}\right)$ and $\left(A_{c_{2}}^{\varphi },\varphi_{c_{2}}^{-1}\right)$ be charts about $p\in P_{\mu }$ and $g:I\subset T\to P_{\mu }$ a curve such that $g(t_{0})=p$, for some $t_{0}\in T$. Taking $g\left(t\right)=\varphi_{c_{1}}\left(u_{1}\right)=\varphi \left(c_{1}+u_{1}-\psi \left(u_{1}\right)\right)$, we have that $u_{1}\left(t\right)=\varphi_{c_{1}}^{-1}\left(g\left(t\right)\right)$. Moreover, $g\left(t\right)=\varphi_{c_{1}}\left(u_{1}\right)$ and $u_{2}\left(t\right)=\varphi_{c_{2}}^{-1}\left(g\left(t\right)\right)$. Using random variables we have that $u_{2}\left(t_{0}\right)=\varphi_{c_{2}}^{-1}\left(g\left(t_{0}\right)\right)=\varphi_{c_{2}}^{-1}\circ \varphi_{c_{1}}\left(u_{1}\left(t_{0}\right)\right)$. Hence, by the chain rule we can write

$$u_{2}^{\prime }\left(t_{0}\right)={\left(\varphi_{c_{2}}^{-1}\circ \varphi_{c_{1}}\right)}^{\prime }\left(u_{1}\left(t_{0}\right)\right)u_{1}^{\prime }\left(t_{0}\right)={\left(\varphi_{c_{2}}^{-1}\circ \varphi_{c_{1}}\right)}^{\prime }\left(\varphi_{c_{1}}^{-1}\left(p\right)\right)u_{1}^{\prime }\left(t_{0}\right).$$

We will denote $\tau (P_{\mu })$ as the tangent bundle, which is defined as the disjointed unity of $T_{p}\left(P_{\mu }\right)$, that is,

$$\tau \left(P_{\mu }\right)=\underset{p\in P_{\mu }}{⨆}T_{p}\left(P_{\mu }\right).$$

**Proposition** **8.** *The local representation of the tangent bundle $\tau (P_{\mu })$ is of the form*

(20) $$\left(u_{1,}v_{1}\right)\mapsto \left(\varphi_{c_{2}}^{-1}\circ \varphi_{c_{1}}\left(u_{1}\right),\;v_{1}-\frac{\int_{T}v_{1}{\left(\varphi \right)}_{+}^{\prime }\left(c_{2}\right)d\mu }{\int_{T}{\left(\varphi \right)}_{+}^{\prime }\left(c_{2}\right)d\mu }\right)\in {\tilde{K}}_{c_{2}}^{\varphi }\times L_{c_{2}}^{\varphi }.$$

**Proof.**  Given $w\in \varphi_{c_{1}}^{-1}\left(A_{c_{1}}^{\varphi }\cap A_{c_{2}}^{\varphi }\right),$ we have that the derivative of the map $\varphi_{c_{2}}^{-1}\circ \varphi_{c_{1}}$ evaluated at *w* in the direction of $v\in L_{c}^{\varphi }$ is of the form

(21) $${\left(\varphi_{c_{2}}^{-1}\circ \varphi_{c_{1}}\right)}^{\prime }\left(w\right)v=v-\frac{\int_{T}v{\left(\varphi \right)}_{+}^{\prime }\left(c_{2}\right)d\mu }{\int_{T}{\left(\varphi \right)}_{+}^{\prime }\left(c_{2}\right)d\mu }.$$

In fact, by the convexity of φ, we have that

$$\int_{T}\left(c_{1}-c_{2}+w\right){\left(\varphi \right)}_{+}^{\prime }\left(c_{2}\right)d\mu \leq \int_{T}\left[\varphi \left(c_{1}+w\right)+\varphi \left(c_{2}\right)\right]d\mu .$$

Since $w\in \varphi_{c_{1}}^{-1}\left(A_{c_{1}}^{\varphi }\cap A_{c_{2}}^{\varphi }\right)\subset {\tilde{K}}_{c_{1}}^{\varphi },$ we have that $\varphi (c_{1}+w)$ is μ-integrable, and consequently, $\int_{T}\left(c_{1}-c_{2}+w\right){\left(\varphi \right)}_{+}^{\prime }\left(c_{2}\right)d\mu$ is μ-integrable. Then, from the dominated convergence theorem follows that (21) occurs.

The tangent bundle is then denoted by

$$\tau \left(P_{\mu }\right)=\left\{\left(\varphi_{c}\left(u\right),v\right);\;\varphi_{c}\left(u\right)\in A_{c}^{\varphi }\subset P_{\mu }\;\mathrm{and}\;v\;\mathrm{is}\;a\;\mathrm{tangent}\;\mathrm{vector}\;\mathrm{to}\;\varphi_{c}\left(u\right)\right\}.$$

Its charts are expressed as

$$\left(v,u\right)\in \tau \left(A_{c}^{\varphi }\right)\mapsto \left(\varphi_{c_{2}}^{-1}\left(v\right),\;v-\frac{\int_{T}v{\left(\varphi \right)}_{+}^{\prime }\left(c_{2}\right)d\mu }{\int_{T}{\left(\varphi \right)}_{+}^{\prime }\left(c_{2}\right)d\mu }\right),$$

which was defined in the collection of open subsets $A_{c_{1}}^{\varphi }\times {\tilde{K}}_{c_{1}}^{\varphi }$ of $P_{\mu }\times L_{c_{1}}^{\varphi }$. Then, since Equation (21) occurs, the transition mappings are given for $\left(u_{1,}v_{1}\right)\in {\tilde{K}}_{c_{1}}^{\varphi }\times L_{c_{1}}^{\varphi }$ by

(22) $$\left(u_{1,}v_{1}\right)\mapsto \left(\varphi_{c_{2}}^{-1}\circ \varphi_{c_{1}}\left(u_{1}\right),\;v_{1}-\frac{\int_{T}v_{1}{\left(\varphi \right)}_{+}^{\prime }\left(c_{2}\right)d\mu }{\int_{T}{\left(\varphi \right)}_{+}^{\prime }\left(c_{2}\right)d\mu }\right)\in {\tilde{K}}_{c_{2}}^{\varphi }\times L_{c_{2}}^{\varphi }.$$

  □

## 5. Divergence in Statistical Manifolds

This section will be divided into two parts. The first one is responsible by the definition of the φ-divergence for the case where φ is the deformed exponential defined in Section 3 and to define a divergence using the *q*-exponential. In the second part, we prove that the *q*-exponential and κ-exponential functions can be used to generalize the divergence of Rényi [13,21].

### 5.1. The φ-Divergence and *q*-Divergence

To define the divergence associated to the normalization function $\psi :\tilde{K}{}_{c}^{\varphi }\to R$ is necessary the convexity of ψ. This is guaranteed by the fact that $N_{c}^{\varphi }$ is a subspace and $\psi :K_{c}^{\varphi }\to R$ is convex [10]. In this way, the Bregman’s divergence $B_{\psi }:{\tilde{B}}_{c}^{\varphi }\times {\tilde{B}}_{c}^{\varphi }\to \left[0,\infty \right)$ associated the $\psi :{\tilde{B}}_{c}^{\varphi }\to \left[0,\infty \right)$ is given by [22,23,24]

(23) $$B_{\psi }\left(v,u\right)=\psi \left(v\right)-\psi \left(u\right)-\partial_{+}\psi \left(u\right)\left(v-u\right).$$

Then, we can define the divergence $D_{\psi }:{\tilde{B}}_{c}^{\varphi }\times {\tilde{B}}_{c}^{\varphi }\to \left[0,\infty \right)$ related the generalized φ-family $A_{c}^{\varphi }$ as $D_{\psi }\left(u,v\right)=B_{\psi }\left(v,u\right)$.

Given $u,v\in {\tilde{B}}_{c}^{\varphi }$, we have that $\varphi \left(c+u-\psi \left(u\right)\right),\;\varphi \left(c+v-\psi \left(v\right)\right)\in P_{\mu }$ and as a consequence $c+u-\psi \left(u\right),\;c+v-\psi \left(v\right)>a_{\varphi }$. Supposing φ is continuously differentiable, it follows that the divergence $D_{\psi }$ does not depend on the parametrization of $A_{c}^{\varphi }$. This allows us to define the divergence between the probability densities $p=\varphi_{c}\left(u\right)$ and $z=\varphi_{c}\left(v\right)$, for $u,\;v\in {\tilde{B}}_{c}^{\varphi }$ as

(24) $$D\left(p\|z\right)=D_{\psi }\left(u,v\right)=\frac{\int_{T}\frac{\varphi_{c}^{-1}\left(p\right)-\varphi_{c}^{-1}\left(z\right)}{{\left(\varphi_{c}^{-1}\right)}^{\prime }\left(p\right)}d\mu }{\int_{T}\frac{1}{{\left(\varphi_{c}^{-1}\right)}^{\prime }\left(p\right)}d\mu }.$$

Note that the divergence is well defined inside the same φ-family. The condition D(p‖z)=∞ if *p* and *z* are not in the same φ-family extends the divergence for $P_{\mu }$. We will denote those divergence by $D_{\varphi }$ and called it φ-divergence [10].

Given $u,\;v\in {\tilde{B}}_{c}^{\varphi }$, we have that $u,\;v>a_{\varphi }$, then φ(t,.) is strictly convex in ${\tilde{B}}_{c}^{\varphi }$, and therefore $D_{\varphi }$ is always non-negative and $D_{\varphi }\left(p\|z\right)$ is equal to zero if and only if p=z. In the following example, we find the φ-divergence for the case in which the deformed exponential function φ is the *q*-deformed exponential function.

**Example** **3.** *Consider the q-exponential $\exp_{q}\left(t,u\right)=\exp_{q(t)}\left(u\right)$ instead of φ(t,u), whose inverse $\varphi^{-1}\left(t,u\right)$ is the q-logarithm $\ln_{q}\left(t,u\right)=\ln_{q(t)}\left(u\right).$ Then, we have*

$$D\left(p\|z\right)=\frac{\int_{T}\frac{\ln_{q}\left(p\right)-\ln_{q}\left(z\right)}{\ln_{q}^{\prime }\left(p\right)}d\mu }{\int_{T}\frac{1}{\ln_{q}^{\prime }\left(p\right)}d\mu },$$

*where $\ln_{q}\left(p\right)$ denotes $\ln_{q(t)}\left(p\left(t\right)\right).$ Since the q-logarithm $\ln_{q}\left(u\right)=\frac{u^{1-q}-1}{1-q},$ has as derivative $\ln_{q}^{\prime }\left(u\right)=\frac{1}{u^{q}},$ we have that*

$$\int_{T}\frac{\ln_{q}\left(p\right)-\ln_{q}\left(z\right)}{\ln_{q}^{\prime }\left(p\right)}d\mu =\int_{T}\frac{\frac{p^{1-q}-1}{1-q}-\frac{z^{1-q}-1}{1-q}}{\frac{1}{p^{q}}}d\mu =\int_{T}\frac{p^{q}\left(p^{1-q}-z^{1-q}\right)}{1-q}d\mu$$

*and*

$$\int_{T}\frac{1}{\ln_{q}^{\prime }\left(p\right)}d\mu =\int_{T}\frac{1}{\frac{1}{p^{q}}}d\mu =\int_{T}p^{q}d\mu .$$

*Therefore*

(25) $$D\left(p\|z\right)=\frac{\int_{T}\frac{p^{q}\left(p^{1-q}-z^{1-q}\right)}{1-q}d\mu }{\int_{T}p^{q}d\mu }.$$

The divergence D(p‖z) in (25) is related with the *q*-divergence defined in (6). In fact,

$$\begin{array}{ll} I^{\left(q\right)}\left(p\|z\right) & =\int_{T}zf\left(\frac{p}{z}\right)d\mu \\ & =\int_{T}z\left(\frac{p}{z}\ln_{q}\left(\frac{z}{p}\right)\right)d\mu \\ & =-\int_{T}p\left(\frac{\ln_{q}\left(z\right)-\ln_{q}\left(p\right)}{1+\left(1-q\right)\ln_{q}\left(p\right)}\right)d\mu \\ & =-\int_{T}p\left(\frac{\left(z^{1-q}-p^{1-q}\right)/\left(1-q\right)}{1+\left(1-q\right)\frac{p^{1-q}-1}{\left(1-q\right)}}\right)d\mu \\ & =\int_{T}p^{q}\left(\frac{\left(p^{1-q}-z^{1-q}\right)}{\left(1-q\right)}\right)d\mu . \end{array}$$

Then $D\left(p\|z\right)=\frac{I^{\left(q\right)}\left(p\|z\right)}{\int_{T}p^{q}d\mu }$ and we can define the metric $g:\Sigma \left(P_{\mu }\right)\times \Sigma \left(P_{\mu }\right)\to F\left(P_{\mu }\right)$ as

(26) $$g\left(u,v\right)=\frac{q\int_{T}\frac{uv}{z}d\mu }{\int_{T}z^{q}d\mu },$$

where $\Sigma (P_{\mu })$ is the set of vector fields $u:A_{c}^{\varphi }\to T_{p}\left(A_{c}^{\varphi }\right)$ and $F(P_{\mu })$ the set of $C^{\infty }$ functions $f:A_{c}^{\varphi }\to R$. This map is well defined, since ${\left(\frac{\partial }{\partial u}\right)}_{p}{D\left(p\|z\right)|}_{p=z}=0$ and ${\left(\frac{\partial }{\partial v}\right)}_{p}{D\left(p\|z\right)|}_{p=z}=0.$

Notice that considering $\int_{T}p^{q}d\mu =1$ we will have that divergence in (25) coincides with the *q*-divergence defined in [15], the metric in (26) coincides with the metric given in [25] and the family of covariant derivatives (connections) given by

$$\nabla_{w}^{q}u=\frac{\partial }{\partial w}u-\frac{\left(1-q\right)}{r}uw+\frac{u}{A^{2}}B-w\frac{C}{A^{2}},$$

where $A=\int_{T}z^{q}d\mu ,\;B={\left(\frac{\partial }{\partial w}\right)}_{p}{A|}_{p=z}$ and $C={\left(\frac{\partial }{\partial u}\right)}_{p}{A|}_{p=z}$ coincides with the family of covariant derivatives (connections) given in [25]. The notation ${\left(\frac{\partial }{\partial w}\right)}_{p}{A|}_{p=z}$ means the derivative of *A* in the direction of *w* in the point *z* when p=z.

### 5.2. Generalization of Divergence of Rényi and $\exp_{\kappa }$

Now, we will recall that the Rényi divergence is related with the φ-divergence and we will see that a necessary and sufficient condition for the existence of generalization of Rényi divergence is the condition (a2). Consequently, we prove that the *q*-deformed exponential and κ-exponential functions can be used in the generalization of Rényi divergence.

In [12] was defined a generalization of the Rényi divergence of order α∈(0,1) as

(27) $$D_{R,\varphi }^{\left(\alpha \right)}\left(p\|z\right)=\frac{\kappa (\alpha )}{\alpha (1-\alpha )},$$

where κ(α) satisfies the Equation (12). This generalization in the case α∈{0,1} is defined as the limit

(28) $$D_{R,\varphi }^{\left(0\right)}\left(p\|z\right)=\lim_{\alpha \to 0}D_{R,\varphi }^{\left(\alpha \right)}\left(p\|z\right)$$

and

(29) $$D_{R,\varphi }^{\left(1\right)}\left(p\|z\right)=\lim_{\alpha \to 1}D_{R,\varphi }^{\left(\alpha \right)}\left(p\|z\right).$$

The limits in (28) and (29), under some conditions, are finite-valued and converges to the φ-divergence:

$$D_{R,\varphi }^{\left(0\right)}\left(z\|p\right)=D_{R,\varphi }^{\left(1\right)}\left(p\|z\right)=D_{\varphi }\left(p\|z\right)<\infty .$$

In the next proposition we have that a necessary and sufficient condition to connect two probability densities of $P_{\mu }$ by an open arc is the condition (a2).

**Proposition** **9** ([12], Proposition 1)**.** *Let μ be a non-atomic measure. Consider φ:R→[0,∞) be a positive, deformed exponential function. Fix any α∈(0,1). The condition (a2) is satisfied if, and only if, given p and z in $P_{\mu }$, there exists a constant κ(α):=κ(α;p,z) such that*

(30) $$\int_{T}\varphi \left(\left(1-\alpha \right)\varphi^{-1}\left(p\right)+\alpha \varphi^{-1}\left(z\right)-\kappa \left(\alpha \right)\right)d\mu =1.$$

In the Example 1, where the measure μ was assumed to be non-atomic, we have that the *q*-exponential function satisfies the condition (a2). Then, by Proposition 9 and Equation (27), we conclude that this function can be used in the generalization of Rényi divergence. Analogously, the function given in the Example 2 cannot be used in the generalization of Rényi divergence.

Supposing that μ is non-atomic, it is presented on the next proposition an equivalent criterion for a deformed exponential function φ to satisfy condition (a2).

**Proposition** **10** ([12], Proposition 3)**.** *Let φ:R→[0,∞) be a deformed exponential function. Then (a2) is satisfied if, and only if,*

$$\underset{u\to \infty }{lim sup}\frac{\varphi (u)}{\varphi (u-\lambda_{0})}<\infty ,\;for\;some\;\lambda_{0}>0.$$

In the next example, we will show a class of deformed exponential functions that can be used in the generalization of Rényi divergence.

**Example** **4.** *We will show that the Kaniadakis κ-exponential $\exp_{\kappa }\left(.\right)$ satisfies the condition (a3). The κ-exponential $\exp_{\kappa }:R\to \left(0,\infty \right)$ for κ∈[−1,1] is defined as [26,27]*

$$\exp_{\kappa }\left(u\right)=\left\{\begin{array}{cc} {\left(\kappa u+\sqrt{1+\kappa^{2}u^{2}}\right)}^{\frac{1}{\kappa }}, & if\;\kappa \neq 0,\\ \exp(u) & if\;\kappa =0. \end{array}\right.$$

*Its inverse, the so called κ-logarithm $\log_{\kappa }:\left(0,\infty \right)\to R$, is given by*

$$\log_{\kappa }\left(u\right)=\left\{\begin{array}{cc} \frac{v^{\kappa }-v^{-\kappa }}{2\kappa }, & if\;\kappa \neq 0,\\ \ln(v) & if\;\kappa =0. \end{array}\right.$$

*We will verify that there exists α∈(0,1) and λ>0 for which*

(31) $$\lambda \leq \log_{\kappa }\left(v\right)-\log_{\kappa }\left(\kappa v\right),\;for\;all\;v>0.$$

*Some manipulations imply that the derivative of $\log_{\kappa }\left(v\right)-\log_{\kappa }\left(\alpha v\right)$ is negative for $0<v\leq v_{0}$ and positive for $v\geq v_{0}$, where*

$$v_{0}={\left(\frac{\alpha^{-\kappa }-1}{1-\alpha^{\kappa }}\right)}^{\frac{1}{2\kappa }}>0.$$

*Consequently, the difference $\log_{\kappa }\left(v\right)-\log_{\kappa }\left(\alpha v\right)$ attains a minimum at $v_{0}$. Given α∈(0,1), inequality (31) is satisfied for some λ>0. Inserting $v=\exp_{\kappa }\left(u\right)$ into (31), we can write*

(32) $$\alpha \exp_{\kappa }\left(u\right)\leq \exp_{\kappa }\left(u-\lambda \right),\;for\;all\;u\in R.$$

*If n∈N is such that nλ≥1, then a repeated application of (32) yields*

$$\alpha^{n}\exp_{\kappa }\left(u\right)\leq \exp_{\kappa }\left(u-n\lambda \right)\leq \exp_{\kappa }\left(u-1\right),\;for\;all\;u\in R.$$

*Then,*

$$\underset{u\to \infty }{lim sup}\frac{\varphi (u)}{\varphi (u-\lambda_{0})}=\underset{u\to \infty }{lim sup}\frac{\exp_{\kappa }\left(u\right)}{\exp_{\kappa }\left(u-1\right)}\leq \underset{u\to \infty }{lim sup}\frac{1}{\alpha^{n}}<\infty .$$

Therefore, by Proposition 10 Kaniadakis κ-exponential $\exp_{\kappa }\left(.\right)$ satisfies the condition (a2).

As consequence of the Example 4 and Proposition 9, we have that $\exp_{\kappa }\left(u\right)$ can be used in the generalization of Rényi divergence.

## 6. Conclusions

In this paper we constructed a parametrization of the statistical Banach manifold using a deformed exponential function. We have found the tangent space of $P_{\mu }$ in *p* and we also constructed the tangent bundle of $P_{\mu }$. We defined the φ-divergence where φ is the *q*-exponential function and we establish a relation between this divergence and the *q*-divergence defined in [15]. Another important contribution is that the *q*-exponential and κ-exponential functions can be used to generalize the divergence of Rényi. The perspective for future works is to define the parallel transport, once we find the tangent plane. We also intend to construct a parametrization for $P_{\mu }$ using a deformed exponential function satisfying (a1) in the case where for each measurable function c:T→R, with $\int_{T}\varphi \left(c\right)d\mu =1$, there exists a measurable function $u_{0c}:T\to R$, such that $\int_{T}\varphi \left(c+\lambda u_{0c}\right)d\mu <\infty$, for each λ>0.
